# Comparative effectiveness of post-discharge interventions for hospitalized smokers: study protocol for a randomized controlled trial

**DOI:** 10.1186/1745-6215-13-124

**Published:** 2012-08-01

**Authors:** Sandra J Japuntich, Susan Regan, Joseph Viana, Justyna Tymoszczuk, Michele Reyen, Douglas E Levy, Daniel E Singer, Elyse R Park, Yuchiao Chang, Nancy A Rigotti

**Affiliations:** 1 Tobacco Research and Treatment Center, Massachusetts General Hospital, Boston, USA; 2 General Medicine Division, Department of Medicine, Massachusetts General Hospital, Boston, USA; 3 Mongan Institute for Health Policy, Partners HealthCare and Massachusetts General Hospital, Boston, USA; 4 Harvard Medical School, Boston, USA; 5 Massachusetts General Hospital, , Boston, MA 02114, USA

**Keywords:** Smoking cessation, Hospitalization, Pharmacotherapy, Counseling, Randomized clinical trial, Interactive voice response

## Abstract

**Background:**

A hospital admission offers smokers an opportunity to quit. Smoking cessation counseling provided in the hospital is effective, but only if it continues for more than one month after discharge. Providing smoking cessation medication at discharge may add benefit to counseling. A major barrier to translating this research into clinical practice is sustaining treatment during the transition to outpatient care. An evidence-based, practical, cost-effective model that facilitates the continuation of tobacco treatment after discharge is needed. This paper describes the design of a comparative effectiveness trial testing a hospital-initiated intervention against standard care.

**Methods/design:**

A two-arm randomized controlled trial compares the effectiveness of standard post-discharge care with a multi-component smoking cessation intervention provided for three months after discharge. Current smokers admitted to Massachusetts General Hospital who receive bedside smoking cessation counseling, intend to quit after discharge and are willing to consider smoking cessation medication are eligible. Study participants are recruited following the hospital counseling visit and randomly assigned to receive Standard Care or Extended Care after hospital discharge. Standard Care includes a recommendation for a smoking cessation medication and information about community resources. Extended Care includes up to three months of free FDA-approved smoking cessation medication and five proactive computerized telephone calls that use interactive voice response technology to provide tailored motivational messages, offer additional live telephone counseling calls from a smoking cessation counselor, and facilitate medication refills. Outcomes are assessed at one, three, and six months after hospital discharge. The primary outcomes are self-reported and validated seven-day point prevalence tobacco abstinence at six months. Other outcomes include short-term and sustained smoking cessation, post-discharge utilization of smoking cessation treatment, hospital readmissions and emergency room visits, and program cost per quit.

**Discussion:**

This study tests a disseminable smoking intervention model for hospitalized smokers. If effective and widely adopted, it could help to reduce population smoking rates and thereby reduce tobacco-related mortality, morbidity, and health care costs.

**Trial registration:**

United States Clinical Trials Registry NCT01177176.

## Background

Smoking cessation has health benefits for all smokers, even those with chronic disease [1-3]. The health care system is an important channel for delivering tobacco treatment to smokers. The 2008 US Public Health Service clinical practice guideline recommends offering treatment, defined as counseling and medication, to all smokers in every health care setting, including hospitals [4]. Smoking status documentation is included as a core measure for the ‘meaningful use’ of electronic health records in a federal government program that offers substantial incentive payments to US physicians and hospitals [5]. This manuscript describes the protocol for a randomized clinical trial that compares the effectiveness of two hospital-initiated tobacco treatment programs that aim to provide post-discharge cessation treatment.

Each year, nearly four million smokers in the United States (8.7% of all smokers in the US) spend at least one night in a hospital [6]. This event provides smokers an opportunity to initiate smoking cessation for several reasons. First, smokers must temporarily abstain from tobacco to comply with smoke-free hospital policies. Second, illness, especially if tobacco-related, boosts motivation to quit [7]. Third, hospitalized smokers have intensive contact with medical personnel who could initiate tobacco treatment. According to a 2007 Cochrane systematic review of 33 randomized controlled trials of hospital-initiated smoking cessation interventions, hospital-based smoking cessation interventions are effective, but only if supplemented with at least one month of follow-up contact after discharge [8]. The review, like the 2008 US Public Health Service Clinical Practice guideline, recommended that all hospitalized smokers should be offered a smoking counseling intervention [4]. Subsequent trials support this conclusion [9-11].

Translation of this research into clinical practice was facilitated in 2004 when tobacco items were included in National Hospital Quality Measures (NHQM) adopted by the Joint Commission (JC) and the US federal government’s Centers for Medicare and Medicaid Services. These require hospitals to report publicly how often smokers with three smoking-related diagnoses (acute myocardial infarction, congestive heart failure, pneumonia) receive smoking cessation advice and counseling [12]. These measures stimulated many hospitals to provide brief smoking cessation interventions. These measures, while helpful, were imperfect. They did not apply to all hospitalized smokers and did not require hospitals to link patients to tobacco intervention after discharge. Consequently, hospitals that met NHQM standards may not have improved long-term smoking cessation rates. To address these limitations, the JC has adopted a revised set of tobacco NHQMs that are currently being reviewed by national stakeholders. The revised measures apply to hospitalized smokers with all diagnoses, require hospitals to document offers of both medication and counseling to smokers and document the offer of a plan for post-discharge counseling and medication [13]. These proposed guidelines have created a need for broadly disseminable, post-discharge smoking cessation interventions.

Sustaining contact and treatment for smokers after hospitalization provides challenges. First, hospitalized smokers, motivated by a serious medical event and perhaps attributing their nicotine withdrawal symptoms to the discomfort of hospitalization, may not anticipate their difficulty staying quit when they return home. Despite high levels of motivation to quit reported in hospitalized smokers [14,15] at least half of smokers, resume smoking within three days of hospital discharge [16]. Second, sustaining treatment regimens from inpatient and outpatient care settings is a challenge for the management of chronic diseases, including tobacco dependence [17-19]. Strategies to avoid gaps in care are needed to prevent relapse after discharge. Third, while hospital clinicians can provide prescriptions for smoking cessation medications, many of these medications are not covered by insurance, leaving barriers of cost and convenience which preclude patients from filling them [20]. Information about the cost effectiveness of post-discharge smoking cessation interventions in terms of reductions in longer-term healthcare spending might encourage insurers to expand coverage. Fourth, while hospital staff can provide information about available community smoking cessation resources for post-discharge care, few patients access these resources if the referral is done passively [21]. This may be due to barriers of cost, convenience, or lack of instrumental support to coordinate services (for example, social workers, nurses).

### The current study

The current study is a randomized controlled effectiveness trial, the Helping Hospital-initiated Assistance for Nicotine Dependence (Helping HAND) Trial, which compares an Extended Care Model designed to address these barriers and facilitate the delivery of the two components of effective tobacco treatment (smoking cessation medication and counseling support) after hospital discharge against Standard Care. This paper describes the protocol for this clinical trial.

To sustain counseling support, the model incorporates interactive voice response (IVR) technology to triage patients to post-discharge treatment resources. IVR is a telephone technology in which a computer detects voice and touch tones and responds to callers with pre-recorded audio. In the hospital setting, IVR systems are used to assess patients for adverse outcomes after outpatient surgery [22-25]. A ‘call-out’ IVR system, in which a computer initiates the call, could reduce the cost of contacting smokers by substituting a computer for a human caller, making multiple calls, and calling outside normal business hours. IVR can serve a number of clinical functions including: (1) providing rapid telephone contact after discharge (at a time when patients are at higher risk for relapse), (2) providing motivational messages and information to encourage patients to continue cessation efforts and medication use, (3) enhancing counseling efficiency by identifying smokers likely to benefit from it, and (4) facilitating successful connection to counseling services.

IVR technology has been used to sustain contact with smokers after hospital admission. The feasibility of an IVR system designed for smokers with coronary heart disease (CHD) called the Ottawa Model was demonstrated in an uncontrolled study [26]. A small pilot trial assessing the model produced a non-significant increase in one-year point prevalence quit rates over usual care, from 35% to 46%, but the trial was underpowered [27]. The Ottawa Model was subsequently expanded for use with all hospitalized smokers and, in a non-randomized pre-post study it was successfully implemented as a comprehensive program for smokers who were admitted to nine general hospitals in Ontario, Canada [28]. Six-month continuous quit rates increased from 18% before to 29% after implementation. That study assessed the combined effect of both the inpatient and outpatient components of the model and therefore was not designed to test the independent contribution of the post-discharge component.

The second component of the Extended Care Model aims to increase adherence to smoking cessation medication after discharge. Patients are given a free one-month supply of their choice of smoking cessation medication and offered two free refills. This obviates filling a prescription or paying for medications not covered by health insurance. The 2008 Clinical Practice Guideline demonstrated that using FDA-approved smoking cessation medication (nicotine replacement therapy, bupropion and varenicline) can double to triple the odds of successful cessation [4]. Providing free medication at discharge, rather than medication recommendations or prescriptions, removes barriers of cost and convenience to increase use [29-31]. Primary care clinics and state tobacco control programs that have provided free medication with few barriers to access have observed increased smokers’ utilization of medication when trying to quit [32-37].

The aim of the Helping HAND trial is to compare the effectiveness of the Extended Care program with Standard Care for increasing smoking cessation rates at six months after hospital discharge. The Extended Care program provides free smoking cessation medication and proactive IVR calls for three months after hospital discharge. The calls assess smoking status, triage patients to smoking cessation resources and facilitate the provision of up to a three-month supply of FDA-approved smoking cessation medication. We hypothesize that Extended Care will increase rates of smoking cessation compared to standard care and will also increase the use of smoking cessation resources (counseling and medication) after discharge. An exploratory aim is to determine the cost-effectiveness of the program, compared to Standard Care, in terms of reduction of healthcare costs due to reduced utilization of hospital-based acute care in the Extended Care group (hospital readmissions and emergency room visits).

## Methods/design

### Design

**Figure 1:**
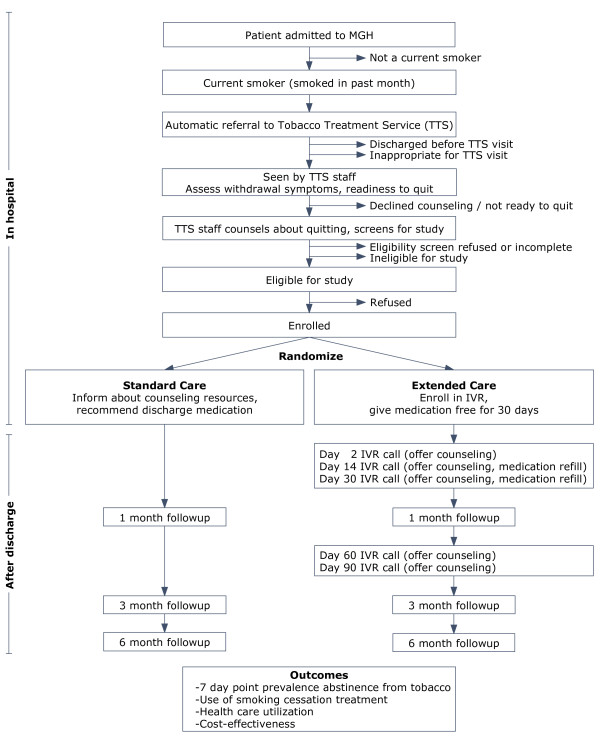
Helping HAND study diagram.

The Helping HAND study is a two-arm randomized controlled trial of a post-discharge treatment program for hospitalized smokers (Figure 1). The arms are Standard Care and Extended Care. Participants are followed for six months after hospital discharge. The study site is Massachusetts General Hospital (MGH), a 900-bed urban teaching hospital in Boston, MA, which admitted 51,024 patients in 2009. The goal is to recruit 330 hospitalized smokers. Recruitment began in July 2010. The study is approved by the Partners Health System Institutional Review Board and is registered with the United States National Institute of Health Clinical Trials Registry (NCT01177176).

### Participants

#### Inclusion criteria

MGH inpatients are eligible for the study if they:

· Are an adult (≥18 years old).

· Are a current daily smoker (smoked ≥ 1 cigarette/day in the past month when smoking in their usual pattern).

· Plan to sustain or initiate a quit attempt after hospital discharge, assessed by asking the smoker to endorse one of four options (‘I will stay quit’, ‘I will try to quit’, ‘I don’t know if I’m going to quit’, ‘I do not plan to quit’). Only those smokers who endorse the first two responses are eligible for study enrollment.

· Have received smoking cessation counseling (for more than five minutes) and post-discharge smoking cessation medication recommendations from the MGH Tobacco Treatment Service staff.

· Are being discharged to home or, if discharged to a rehabilitation facility, have a mobile telephone on which they can receive the IVR calls.

#### *Exclusion criteria*

Patients are excluded if they:

· Have cognitive impairment that precludes providing consent or participating in the intervention (for example, dementia, delirium, active suicidal ideation, or other altered mental status).

· Have a communication barrier precluding use of interactive voice response calls (for example, speech or hearing impairment, unable to speak English).

· Are pregnant, are too sick to receive the intervention or have limited life expectancy (for example, metastatic cancer, on hospice or palliative care; admitted from a nursing home).

· Have an admitting diagnosis directly due to active substance abuse (for example, overdose, detox) or have a past year history of substance use disorder diagnosis (excluding marijuana, alcohol and tobacco).

· Are unwilling to accept smoking cessation medications to take home.

### Procedure

#### *MGH inpatient protocol for smokers*

The smoking status of all patients admitted to MGH is identified on admission and documented in the hospital’s computerized order entry system. This system includes a drop-down menu with a check box for ordering nicotine patches. All FDA-approved medications, including the nicotine patch, gum, lozenge, and inhaler, bupropion, and varenicline, are on the hospital formulary. An internally-developed four-page pamphlet, *A Guide for Hospital Patients Who Smoke,* is put in the admission packet at every new patient’s bedside. It outlines reasons why a hospital admission is a good time to quit, educates patients about nicotine withdrawal symptoms and how to manage them, and provides contact information for community smoking cessation resources, including the state quitline.

The computerized order entry system automatically delivers to the MGH Tobacco Treatment Service (TTS) an electronic list of all admitted patients identified as having smoked in the past year. A TTS staff member (a certified Tobacco Treatment Specialist) [38] attempts to visit each smoker at the bedside, usually on the day after admission. The counselor follows a protocol to maximize patient comfort by ensuring adequate treatment of nicotine withdrawal symptoms in the hospital, provides advice to quit, and assesses a smoker’s readiness to quit after discharge. For smokers who are not ready to quit, these visits are brief, typically lasting fewer than five minutes, and these individuals are not eligible for study recruitment. For smokers considering tobacco abstinence after hospital discharge, the TTS counselor conducts a standard assessment and helps the smoker to make a quit plan. This includes a recommended smoking cessation medication and information about community smoking cessation counseling. The counselor’s medication recommendation is based on patient medical history, prior experience with cessation medications, and patient preference. It avoids medications contraindicated for the participants’ medical condition or those that have resulted in past adverse reactions. Counseling visits last a median of 20 minutes.

#### *Study recruitment*

The TTS counselor screens inpatients who were counseled (operationally defined as receiving more than five minutes of contact) for inclusion criteria, using a standard form. Patients who meet these criteria are briefly introduced to the study and are referred to research staff, who assess for exclusion criteria using chart review, patient interview, and, as necessary, discussions with the patients’ care team. Eligible patients are provided detailed study information. If willing to participate, they provide written informed consent and complete a ten-minute interview to collect baseline measures and detailed contact information.

#### *Assignment to treatment group*

Participants are randomly assigned at the bedside to either Standard Care or Extended Care by research staff, in a 1:1 fashion in permuted blocks of eight randomization numbers. Randomization is stratified by two expected predictors of smoking cessation success, daily cigarette consumption in the month prior to admission (< 10 versus > = 10 cigarettes per day) and admitting service (cardiac versus other). Randomization is concealed by placing information about randomization condition into sealed envelopes. When a participant is randomized, the research staff member opens the next envelope corresponding to the participant’s randomization stratum, shares it with the participant, and initiates the assigned intervention. Research staff do not track the beginning and end of randomization blocks. Thus, neither the participant nor the research staff know participants’ randomization group before participants are enrolled.

#### *Outcome assessment*

Participants are contacted by study staff via telephone at one, three and six months after discharge to collect outcome measures, including: smoking status, use of smoking cessation medication and counseling, hospital readmissions, emergency room visits and, in the intervention group, satisfaction with the IVR system. A $20 gift card is provided for each completed follow-up call. A reminder letter is sent one month before the six-month call. At one month, the call window begins three days before the target date and ends two weeks after the target date. At three and six months, the call windows begin two weeks before the target date and end four weeks after the target date. Study staff make up to ten call attempts to the participant, using all available phone numbers (for example, home, work, mobile) and calling at several different times of day, with more calls made during the participants’ self-reported best time to call. If participants cannot be reached, study staff call participants’ alternative contacts (people whom the participants reported would know how to reach them) to verify participants’ contact information. A letter is also sent to the participants indicating that we are trying to reach them and asking them to call the study line.

#### *Validation of tobacco abstinence*

Self-reported tobacco abstinence is validated in two ways at three and six month follow-ups: biochemically, and by asking a ‘proxy’ (a friend or family member of the participant) to confirm smoking status. We biochemically validate abstinence status for all participants reporting non-smoking, but we also contact proxies for all participants. First, participants who self-report being quit are asked to mail a saliva sample for assay of cotinine, a nicotine metabolite. Because nicotine replacement therapy (NRT) use produces detectable cotinine, the validation criterion for a patient reporting current NRT use is an expired-air carbon monoxide measurement of ≤9 ppm, obtained at a hospital or home visit [39]. Participants are paid $50 for providing a sample for biochemical validation. We also validate smoking status by proxy report. At the baseline survey participants are asked to give the names of three people who would know whether they were smoking to serve as proxies. At three and six months, after the participant’s self-report is obtained, we call the participant’s proxy to obtain confirmation of the participant’s current smoking status. This is done for participants who report smoking as well as those reporting being abstinent. To ensure that proxies are good reporters of smoking status, we also ask when they last had contact with the participant.

### Interventions

#### *Standard care*

Participants receive the MGH inpatient protocol during the hospital stay. As described above, the smoking counselor provides information to smokers about post-discharge counseling resources and makes a specific recommendation for post-discharge medication. The counselor also puts these recommendations in a written consultation note in the hospital chart.

#### *Extended care*

Participants in the Extended Care group receive standard care for smoking cessation plus a two-component intervention which consists of: (1) a one month supply of smoking cessation pharmacotherapy (that can be refilled twice over the three month intervention period) and (2) five calls from the IVR system (plus opportunities to speak with a live counselor).

##### Pharmacotherapy

A free one-month supply of the counselor-recommended FDA-approved smoking cessation medication is prescribed by the hospital physician, filled in the hospital pharmacy and delivered to the patient by study staff prior to discharge, along with information on proper medication use, side effects, and instructions on how to obtain two free refills using the IVR system described below. Pharmacotherapy choices include single agents (NRT, bupropion, or varenicline) or combination treatments (nicotine patch + gum or lozenge, bupropion + nicotine patch, gum, or lozenge). Information about the discharge medication is sent to the patient’s primary care provider, whom the patient is told to contact in case of adverse events.

##### IVR

The IVR system (administered by TelAsk Technologies, Ottawa, Canada) provides automated telephone calls at 2, 14, 30, 60 and 90 days after discharge. Discharge dates and participant phone numbers are transferred to the IVR vendor by the study team via secure FTP. The IVR system makes up to eight attempts to reach participants for each scheduled call, beginning on the scheduled call day and proceeding with two calls per day for four days or until the call is completed. Calls are scheduled at participants’ preferred times. The current system is adapted from a previous IVR system that was tested in a pilot trial and demonstrated to be feasible [40].

**Figure 2:**
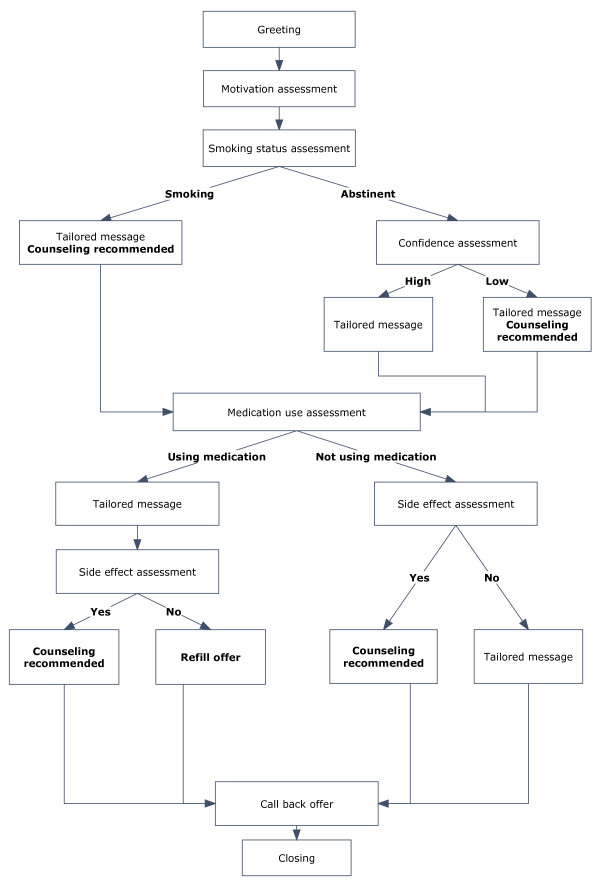
Sample Interactive Voice Response call design.

The IVR calls have four goals: (1) assessing smoking status and medication use, (2) providing motivational messages tailored to smoking and medication use status, (3) triaging participants to smoking cessation resources, and (4) offering refills of the study medication. Each IVR call assesses current smoking status and intention to quit, current use of smoking cessation medication, medication side effects, and patient’s desire for telephone counseling support. This information is used to tailor motivational messages that encourage participants to use smoking cessation medications and to speak with counselors when needed (for example, ‘Using medicine doubles your chance of quitting smoking for good. We recommend that you talk with our tobacco counselor about how to get started on your medicine’). Participants are prompted by the IVR system to renew smoking cessation medications, to change administration schedules when necessary (that is, tapering nicotine replacement therapy), and to speak to smoking cessation counselors when they are having difficulties with medication or with staying quit. While participants can request a return call from a counselor at the end of any IVR call, the system recommends that the participant request a return call if the participant is at high risk for relapse. Criteria to trigger the system to recommend a return call from a live counselor include: (1) participants who do not start pharmacotherapy after discharge or stop pharmacotherapy before the end of the 90-day course; (2) participants who resume smoking after discharge but still want to quit; (3) participants who are quit but have a low level of confidence in their ability to stay quit; (4) participants reporting medication side effects (see Figure 2 for sample IVR call). The IVR system prompts the participant to request a refill at only two calls; however, participants may request refills during any return call from the study counselor.

##### IVR return calls

The smoking cessation counseling is protocolized, uses a motivational interviewing style [7] and focuses on providing behavioral techniques to help participants stay quit or re-start quit attempts and increasing adherence to smoking cessation medication. Behavioral counseling modules include managing withdrawal symptoms, craving, mood and stress, and accessing community resources for smoking cessation treatment. Medication management modules focus on medication instructions, managing side effects, and deciding when to stop medicine or change dose. Participants requesting refills are evaluated for side effects and all medication questions are answered.

### Measures

#### *Baseline*

Demographics are collected by chart review (gender, age, health insurance) and in the baseline survey (race/ethnicity, education, employment status, marital/partner status).

**Table 1:** Helping HAND trial measures by assessment time point

**Measure**	**Baseline**	**Outcomes**		
		**1 Month**	**3 Month**	**6 Month**
Demographics	X			
Smoking History	X			
Nicotine dependence (FTND)	X			
Depression symptoms (CES-D)	X			
Alcohol use (AUDIT-C)	X			
Smoking status	X	X	X	X
Non-cigarette tobacco use	X	X	X	X
Biochemical validation^a^			X	X
Proxy validation^b^			X	X
Smoking during index hospital stay		X		
Readiness to quit smoking		X		
Confidence in ability to quit or stay quit		X		
Smoking cessation medication used		X	X	X
Smoking cessation counseling used		X	X	X
Healthcare utilization				
Hospital admission		X	X	X
Emergency room visit		X	X	X
Satisfaction with IVR^c^		X		

^a^Requested from self-reported nonsmokers only. Saliva cotinine requested by mail, unless participant is using nicotine replacement, in which case a visit for measurement of expired air CO is requested. ^b^Obtained from all participants, regardless of self-reported smoking status at outcome assessment. ^c^If the participant was not reached for the one-month follow-up, these questions were asked at the first follow-up for which the participant was reached. AUDIT-C, Alcohol Use Disorders Identification Test – Consumption; CES-d, Center for Epidemiological Studies 8-item Depression Scale; FTND, Fagerström Test for Nicotine Dependence; IVR, Interactive Voice Response.

Smoking history is collected during the hospital tobacco counseling visit and includes: cigarettes per day in the month prior to admission, use of non-cigarette tobacco products, age of smoking initiation, number of years smoking, quit history (ever tried to quit, length of longest quit), previous use of tobacco treatment (counseling, pharmacotherapy), living with a smoker, home smoking policy, in-hospital rating of cigarette craving, perceived importance of quitting, perceived confidence in ability to quit (ten point Likert scales), and intention to quit upon discharge from the hospital (‘I will stay quit’ or ‘I will try to quit’).

Nicotine dependence is measured using the Fagerström Test of Nicotine Dependence (FTND) [41]. The FTND comprises six items with scores ranging from 0 to 10; higher scores indicate greater dependence.

##### Depression

Depressed mood is measured using the eight-item Center for Epidemiological Studies Depression Scale [42] at baseline. Participants rate depressive symptoms over the past week on a four-point Likert scale. Scores range from 0–24, with higher scores indicating greater depression.

Alcohol use is assessed at baseline with the three-item Alcohol Use Disorders Identification Test – Consumption (AUDIT-C), which assesses quantity and frequency of alcohol use and frequency of binge-drinking episodes and generates a tailored risk rating [43].

##### **Hospital admission characteristics**

Length of hospital stay, primary and secondary hospital discharge diagnoses, hospital service, and patient’s disposition after discharge (home or rehabilitation facility) will be obtained from hospital records.

#### *Follow-up (one, three and six months)*

##### Smoking status

The primary outcome is validated seven-day point prevalence abstinence from tobacco at six months after discharge. The principal secondary outcome measure is self-reported seven-day point prevalence abstinence at six months after discharge. Self-reported tobacco abstinence is defined as answering no to two questions: ‘In the past seven days have you smoked a cigarette, even a puff?’; ‘In the past seven days, have you used any tobacco product other than cigarettes such as cigars, pipes, snuff or chew?’ Patients who report tobacco abstinence are asked to provide biochemical and proxy validation (described above) for the primary outcome measure. Subjects who self-report smoking or whose cotinine or CO measures exceed the cut-offs (10 ng/ml for cotinine; 9 ppm for CO) are counted as smokers. Subjects with missing data (who are lost to follow-up or who self-report nonsmoking but do not provide a sufficient saliva sample, CO measurement, or proxy validation) are considered smokers for the primary analysis, but in secondary analyses, outcome will be imputed via statistical modeling.

Other secondary outcome measures of smoking status include: (1) self-reported seven-day point-prevalence tobacco abstinence at one and three months; (2) continuous abstinence from cigarettes and other tobacco (‘Since you left the hospital, have you smoked a cigarette, even a puff?’; ‘Since you left the hospital, have you used any tobacco product other than cigarettes such as cigars, pipes, snuff or chew?’), (3) prolonged abstinence, defined as not using tobacco at one, three and six month follow-up points; (4) duration of tobacco abstinence after discharge; (5) proportion of participants who make a 24-hour quit attempt after discharge (‘Since you left the hospital have you not smoked for 24 hours because you were trying to quit?’).

##### Stage of change/confidence

We assessed confidence (‘On a scale of one to ten, how confident are you that you will stay quit for the next year, with one being not at all confident and ten being very confident?’) among those who have quit or who have a specific plan to quit in the next 30 days. For those who are still smoking, we assess stage of change by asking if participants have ‘serious plans to quit in the next six months’ and ‘a specific plan to quit in the next 30 days’ [44].

##### Inpatient experience with tobacco abstinence and smoking cessation medication use

At the one-month follow-up (or the first follow-up completed), participants rate the difficulty that they had maintaining abstinence in the hospital (‘How hard was it not to smoke while you were in the hospital?’), using a four point Likert scale (not at all-very). Smoking while in the hospital is assessed (‘Did you smoke a cigarette, even a puff, during your stay in the hospital?’). Finally, we assess use of smoking cessation medications during hospitalization including use of the nicotine patch, nicotine gum, nicotine lozenge, nicotine inhaler, bupropion or varenicline.

##### Smoking cessation medication use/adherence

We assess post-discharge use of any of the FDA-approved smoking cessation medications including nicotine replacement (patch, gum, lozenge, inhaler, nasal spray), bupropion or varenicline. For those medications that are used we assess dose, frequency, duration of use and reason for termination. We also assess the source of these medicines (for example, provided by the study, obtained by prescription or purchased over the counter).

##### Post-discharge smoking cessation counseling use

Participants are asked whether they have spoken to a counselor or health care provider about their smoking since hospital discharge. Those who have spoken to a provider about their smoking are asked to whom they spoke (study counselor, state quitline, doctor, nurse, community counselor), how many times they received smoking cessation counseling, and the main topics discussed.

##### Post-discharge utilization of acute health care services

We assess emergency room visits and hospital readmissions during the six months after the discharge from the hospitalization at which enrollment occurred. Emergency room visits and hospitalizations are assessed two ways. First, an electronic medical record tracking system automatically notifies study staff whenever any participant is admitted to any emergency room or hospital in the Partners HealthCare System, the integrated health care delivery system to which MGH belongs. Second, participants are asked at each follow-up survey whether they have been to an emergency room or been hospitalized since their index hospital discharge. Questions are adapted from the 2008 National Health Interview Survey (‘Were you seen in an emergency room but not admitted to the hospital during the six months after you left MGH on (discharge date)?’; ‘Were you hospitalized anywhere overnight during the six months after you left MGH on (discharge date)? Do not include an overnight stay in the emergency room.’). These allow us to identify a hospitalization or emergency room visit made by a participant to a hospital outside Partners system. We request discharge summaries for admissions to hospitals outside Partners HealthCare System. Measures for health care utilization after discharge consist of: (1) number of hospital readmissions; (2) number of hospital days; (3) number of emergency room visits; (4) total number of hospitalizations and emergency room visits.

##### Satisfaction with IVR system

Participants in the Extended Care group who have answered at least one IVR call are asked three questions about satisfaction with the IVR (‘How helpful was it to get phone calls to check in about your smoking after you left the hospital?’ [very helpful-not at all helpful]; ‘If a friend or family member who smokes and wanted to stop were hospitalized, would you recommend that they be followed by an automated telephone support system to help them stop smoking?’ [strongly recommend-strongly not recommend]; and an open-ended question asking what was and was not helpful about the IVR).

#### *Quality assurance*

All referrals to the inpatient TTS and their disposition are recorded and monitored. Counselor screening forms and referrals are monitored to ensure that appropriate patients are being referred. Screening and recruitment reports are monitored weekly. Research staff meets with study counselors monthly to discuss any problems. Receipt of medication prior to discharge is tracked for each participant, and mailing medication used as the back-up strategy. The IVR web interface and IVR call completion rates are monitored weekly. All counseling sessions are recorded and 10% are reviewed for protocol adherence by a supervisor, a tobacco treatment counselor who is distinct from the one who delivered the treatment. Completion rates of follow-up assessments are reviewed weekly.

### Data analysis

#### *Sample size and power calculations*

We estimate that the primary outcome, verified seven-day tobacco abstinence rate at six-month follow-up, will be 20% among the Standard Care group and 35% among the Extended Care group. With a sample size of 165 per group, the study has 83% power to detect this difference with a 0.05 two-sided significance level. The estimate of the Standard Care cessation rate was extrapolated from three-month outcome data that was routinely collected from the MGH TTS. The rate ratio (35/20 = 1.75) was based on results of a recent meta-analysis [8] but increased to reflect expected improvement due to project innovations.

#### *Comparison of smoking status outcomes*

The primary analysis will be done using the intention-to-treat perspective, regardless of whether patients in the Extended Care group took medications or participated in the IVR intervention. In the primary analysis, patients who are not reached for follow-up or who have not validated smoking cessation (either a saliva cotinine < 10 ng/ml, CO < 9 ppm or proxy verification) will be considered to be smoking. A secondary analysis will use multiple imputation techniques for missing biochemical verification values. Cross-sectional analyses will be conducted for outcomes assessed at one, three and six months after discharge.

Chi-square tests will be used to compare smoking cessation rates between the Extended Care group and the Standard Care group at each time point. Logistic regression analysis will be used to compare the two groups with age, sex, discharge diagnosis (cardiac versus other) and cigarettes smoked per day as the additional factors in the model. A longitudinal analysis using Generalized Estimating Equations (GEE) techniques will be used to assess the overall impact including data from all follow-up times while accounting for similarity in patient data across multiple assessment points. In a secondary analysis, we will use multiple imputation techniques for missing biochemical or proxy validation data. Self-reported seven-day point prevalence abstinence is the principal secondary endpoint.

Other smoking status outcomes compared between groups will be: (1) continuous abstinence; (2) prolonged abstinence (defined as quit at one, three and six months); (3) time to slip or relapse (first cigarette smoked after discharge); and (4) making a quit attempt (defined as intentionally not smoking for ≥ 24 hours) after discharge. Continuous and sustained abstinence and whether or not the participant made a quit attempt will be assessed using chi-square tests and logistic regression. Time to relapse will be assessed using survival analysis with Cox proportional hazards models. A secondary hypothesis is that participants in the Extended Care group will have higher rates of using smoking cessation pharmacotherapy and counseling than the Standard Care group. Use of pharmacotherapy and counseling will be assessed using chi-square tests.

#### *Utilization of acute health care services*

We hypothesize that the Extended Care group will have a lower rate of acute health care utilization than the Standard Care group in the six months after hospital discharge. Logistic regression will be used to determine whether the Standard Care group was more likely to have any hospital admissions or emergency room visits and Poisson regression models will be used to determine whether the Standard Care group had a higher number of admissions or hospital days [45].

#### *Cost effectiveness*

Cost-effectiveness analyses will be assessed by cost per quit as the main outcome to ensure maximum comparability with other published studies [46,47]. The incremental costs per quit are estimated as follows: (Total costs of Extended Care - Total costs for Standard Care)/(Total successful quits at six months for Extended Care - Total successful quits at six months for Standard Care). The major costs tracked in the study are described in Table 2. In the intervention arm, these include Tobacco Counselor time (based on hourly wage), the cost of the IVR service, the cost of providing a free pharmacotherapy to participants after discharge, and the cost of any additional counseling or pharmacotherapy used by participants. The major costs in the Standard Care arm that require tracking are the use of post-discharge counseling or pharmacotherapy. For both study arms we will track the portion of counseling and therapy costs (exclusive of the 90-day supply of pharmacotherapy specific to the intervention) paid for by participants out of pocket versus by insurance. Prospective collection of cost information (for example counselor time) will maximize the accuracy of our data.

**Table 2:** Cost data collection domains

**Resource**	**Description**	**Source of resource use data**	**Source of cost data**
Tobacco counselor time – pre-discharge	Initial assessment (Counselor chart review and meeting with the patient to determine a quit plan), medication recommendations, and counseling	Study records	Human resources data
Tobacco counselor time – post-discharge	Medication and cessation support, time spent trying to reach subjects	Study records, survey responses	Human resources data, published reports and datasets
IVR service	Automated telephone outreach to smokers.	Study records	Study records
Smoking cessation medications	FDA-approved smoking cessation medications provided to patients at discharge from the hospital and subsequent refills (includes mailing costs)	Study records	Hospital billing data
	Smoking cessation medications obtained by patients after discharge	Survey responses	Red Book 2010 [48]

## Discussion

Hospital admission is a ‘teachable moment’. Strong evidence supports the efficacy of smoking interventions for hospitalized patients, but only if smoking treatment is sustained for longer than one month after discharge. For dissemination into practice, the critical challenge is identifying a strategy to link hospitalized smokers to tobacco treatment after discharge. Our study compares two strategies to accomplish this goal.

If the intervention is effective, its sustainability will be an important consideration. An IVR system is potentially sustainable because it has a low per-user cost due to automation of calls that allows counseling to be targeted more efficiently than if a live caller were calling every patient. However, participants who request counseling at the IVR contact need to be contacted by a live counselor at a separate time. The choice of who would make and pay for these calls would affect its sustainability. One strategy to limit costs to the hospital and enhance disseminability would be a system in which participants who request a counseling call are automatically and seamlessly transferred from the IVR system to the state telephone quitline instead of relying on hospital counseling resources.

We hypothesize that providing medication in hand at discharge at no cost to the patient is a critical component of the intervention. This study tests the feasibility and impact of the strategy. If successful, providing free medication will be an added cost in the dissemination of the program because nonprescription nicotine replacement is not covered by many health insurance plans. However, new models of health care organization with different reimbursement strategies, such as accountable care organizations, are being implemented [49]. These organizations may be more amenable to considering coverage of NRT after hospital discharge, especially if this intervention is shown to reduce hospital readmissions or subsequent emergency room use.

In designing the intervention, we considered whether to target all hospitalized smokers or only those who state an intention to quit smoking after discharge. We chose the latter approach, reasoning that less motivated smokers were unlikely to use the interventions, that the intervention would be most cost-effective for those intending to quit, and therefore a more targeted intervention would have the best chance of being adopted into clinical practice. This will likely increase cessation rates in both groups. While we believe that smokers planning to quit are the patients most likely to accept and benefit from our intervention, if it is successful, future studies could implement the intervention more broadly.

Our study design assesses the combined effect of an intervention package consisting of two components (IVR + medication provision) compared to standard care (counseling information and medication recommendation). This design does not permit us to determine the relative contribution of the individual intervention components (IVR/counseling or medication) to any effect that we might find. While a factorial design would allow us to distinguish the independent effects of counseling and medication, this would require a larger sample size. If the combined intervention proves effective, subsequent trials could examine the relative contributions of the counseling support and medication.

In summary, an evidence-based, cost-effective intervention model that is readily adoptable by US hospitals is needed in order to realize the potential impact of hospital-initiated smoking interventions and to meet new hospital quality measures under review. This trial is testing an intervention designed to meet this need. A cost-effective smoking intervention model for hospitalized smokers could, if widely adopted, help to reduce population smoking rates and thereby contribute to reducing tobacco-related mortality, morbidity, and health care costs.

### Trial status

Recruitment was completed in April 2012. Follow-up data collection will be completed in November 2012.

## Abbreviations

CHD: coronary heart disease; GEE: generalized estimating equations; Helping HAND: Helping Hospital-initiated Assistance for Nicotine Dependence; IVR: Interactive Voice Response; MGH: Massachusetts General Hospital; NHQM: National Hospital Quality Measures; NRT: nicotine replacement therapy; TTS: Tobacco Treatment Service.

## Competing interests

Dr. Rigotti has served as an unpaid consultant for Pfizer, Inc., and Free & Clear, Inc., regarding smoking cessation. Her institution has been funded by Nabi Biopharmaceuticals, Inc., to conduct trials of an investigational smoking cessation product. Drs. Singer, Japuntich, Regan, Levy, Park, Chang, Mr. Viana, Ms. Tymoszczuk, and Ms. Reyen have no conflicts of interest related to this project to report.

## Authors’ contributions

All authors contributed to the design of the study. SJ and NR drafted the manuscript. All authors read and approved of the final manuscript.
